# Heavy Metals in Surface Sediment of Plateau Lakes in Tibet, China: Occurrence, Risk Assessment, and Potential Sources

**DOI:** 10.3390/toxics11100804

**Published:** 2023-09-23

**Authors:** Qiongyuan Su, Asfandyar Shahab, Liangliang Huang, Muhammad Ubaid Ali, Yanan Cheng, Jiahuan Yang, Hao Xu, Zhicheng Sun, Qi Zou, Zhongbing Chen, Bin Kang

**Affiliations:** 1 College of Environmental Science and Engineering, Guilin University of Technology, Guilin 541004, China; 2 Guangxi Collaborative Innovation Center for Water Pollution Control and Water Safety in Karst Areas, Guilin 541004, China; 3 Guangxi Key Laboratory of Environmental Pollution Control Theory and Technology, Guilin 541004, China; 4 Department of Soil Sciences, Southern Federal University, 344006 Rostov-on-Don, Russia; 5 College of Fisheries, Ocean University of China, Qingdao 266100, China; 6 School of Public Health and Health Management, Gannan Medical University, Ganzhou 341000, China; 7 Department of Applied Ecology, Faculty of Environmental Sciences, Czech University of Life Sciences Prague, Kamýcká 129, 16500 Prague, Czech Republic

**Keywords:** heavy metals, sediment in Tibetan lakes, risk assessment, natural contribution, anthropogenic activities

## Abstract

Tibetan Plateau lakes have high ecological value and play a crucial role in maintaining ecological balance. This research aimed to study the pollution characteristics, ecological risk, and potential sources of eight heavy metals (As, Cd, Cr, Cu, Hg, Ni, Pb, and Zn) in the surface sediments of 12 Tibetan Plateau lakes. The results of the toxicity risk index (TRI) showed that only Gongzhu Tso (28.09) and La’ ang Tso (20.25) had heavy metals that could pose a very high risk of toxicity to aquatic organisms. Hg posed the highest potential ecological risk to aquatic organisms. Based on the results of multiple analyses, we inferred that the contents of Cr, Cu, Hg, and Ni in sediments of Tibetan lakes were influenced by industrial and agricultural development; Cd, Pb, and Zn were influenced by transport and atmospheric transport; and As was derived from geothermal activity and rock weathering.

## 1. Introduction

Lakes are the interconnection point of the interaction of the various circles of the Earth’s surface system, are an important part of the terrestrial hydrosphere, closely related to the biosphere, atmosphere, lithosphere, etc., and have the function of regulating the regional climate, improving the regional environment, maintaining river runoff to keep the balance of the region, providing water for production, living and agricultural irrigation, and reproducing aquatic organisms. Lakes have produced great economic benefits while maintaining ecological balance [1,2]. Lake sediments are an important source and sink for heavy metals in lakes [1]. The accumulation of heavy metals in lakes seriously affects the safety of lake water quality, and it has been shown that nearly 80% of the world’s population faces water safety problems caused by heavy metal contamination of sediments as a result of human activities [3].

There are more than 2800 lakes with an area of more than 1 km^2^ in China, with a total area of about 80,000 km^2^ [4]. The diversity of geomorphological forms and the remarkable differences in climate and regional socio-economics make the sediments of China’s inland lakes contain extremely rich environmental information. Still, they also have their unique environmental problems [5,6,7]. Over the past few decades, the rapid economic development of China, the increasing production levels of industrial enterprises, and the improvement of human living conditions have resulted in the accumulation of a large number of inorganic and organic pollutants in the ecosystems of lakes in a variety of ways [7,8,9]. Due to their long-term stability, bioaccumulation, and biotoxicity [10], heavy metals often enter the food chain through water bodies and lower organisms, thereby disrupting the normal physiological metabolic activities of organisms and ultimately endangering human health and the ecological environment through bioconcentration [11,12,13]. As, Cu, Cr, Ni, and Zn are essential elements in the composition of living organisms, but these elements can be toxic to living organisms above a specific dose. Cd, Hg, and Pb are non-essential elements for biological metabolic activities and can be harmful to organisms at low concentrations [14,15,16]. For example, long-term exposure to As can cause cardiovascular disease [17,18]. Hg accumulation in the body can lead to immune disorders and kidney failure [19]. The excessive intake of Cu can damage the central nervous system, and Cr may cause tumors of respiratory organs [20]. Cd can cause kidney failure and the chance of lung cancer after accumulating in the body in certain amounts [21]. Pb intake can damage the human somatic nervous system, leading to symptoms such as insomnia and headaches [22,23], while Zn may lead to reduced fertility [24]. The United States Environmental Protection Agency (USEPA) has listed heavy metals such as As, Cd, Cr, Cu, Hg, Ni, Pb, and Zn as priority pollutants to be controlled [25,26], while the pollution of heavy metals in lakes has been receiving increasing attention from researchers and scholars since 2011 when the State Council of China promulgated the National 12th Five-Year Plan for Comprehensive Prevention and Control of Heavy Metal Pollution (2011–2015) [27].

The unique geographical features of the Tibetan Plateau have attracted increasing attention from researchers as global research on lake sediments progresses. Many lakes larger than 1 km^2^ have been formed due to crustal movements, glacial effects, and other effects in the Qinghai–Tibet Plateau [28]. Highland lakes are significant for ecosystems in terms of changes in water environment capacity, water column geochemical cycling of water and sediment biogenic elements, and the pollution of sediment with heavy metals [29]. Although the secondary and tertiary sectors in Tibet are underdeveloped [30], the development of industry, commerce, and tourism in Tibet, through national development strategies such as the development of the western region in China, has improved the production and living conditions of the local people. However, anthropogenic discharges of pollutants into water bodies have contributed to the enrichment of sediments in water bodies. At the same time, recent studies have found that heavy metals in Qinghai Lake sediments were mainly derived from anthropogenic inputs to the atmosphere [31]. It has also been suggested in studies of Brahmaputra sediments that heavy metal elements have little anthropogenic influence but are related to the elemental content in the upper crust [32]. The heavy metals can adhere to the surface of particles and then be transported over long distances by atmospheric transport from eastern China and Southeast Asia, such as India to Tibet, and settle into water bodies [33,34]. In addition, recent studies have shown that climatic temperature and precipitation influence the deposition of both heavy metals and nutrients. Higher climatic temperatures lead to higher TOC concentrations, which affects the deposition of heavy metals. Warmer temperatures may also drive secondary emissions of mercury [35]. Otherwise, geothermal activity can also affect the content of heavy metals in sediments [36].

At present, China’s lake sediment pollution is becoming increasingly complicated, and it is gradually changing from single heavy metal pollution to compound pollution with multiple heavy metals. In addition, researchers in China are still focusing their research on heavy metals in water sediments in typical coastal and inland lakes, while fewer studies have been reported on the sediments of Tibetan lakes in remote areas of China. However, there is a need for more information regarding the heavy metals of sediments over the plateau lakes in Tibet. Therefore, this study aimed to (1) clarify the concentration of As, Cd, Cr, Cu, Hg, Ni, Pb, and Zn in the surface sediments of 12 plateau lakes in Tibet to determine their spatial distribution and pollution characteristics; (2) assess the current ecological risk of heavy metals in the surface sediments of 12 plateau lakes in Tibet; and (3) speculate on the potential sources of heavy metals in the sediments of plateau lakes in Tibet.

## 2. Materials and Methods

### 2.1. Study Area

**Figure 1:**
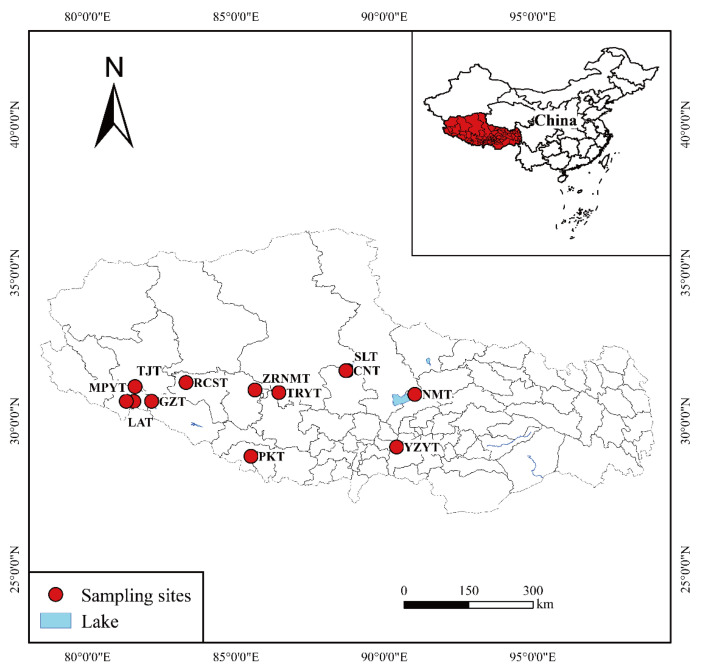
Location map of 12 plateau lake sampling sites in Tibet, China.

The Qinghai–Tibet Plateau, being known as the “third pole” of the world, has the highest elevation, largest area, and most plateau lakes on earth, featuring a concentration of saline lakes [37]. Tibet is located in the southwest of the Tibetan Plateau (26°50′~36°53′ N, 78°25′~99°06′ E) and consists of four major geomorphological regions: the Eastern Tibetan Valley, the Southern Tibetan Basin Valley, the Northern Tibetan Plateau, and the High Himalayan Mountains [38]. A total of 12 lakes on the Tibetan Plateau (28°47′48″~31°41′43″ N, 81°18′27″~91°1′53″ E, Figure 1) were studied in this study, namely Yamzho Yum Tso (YZYT) (Tso, meaning lake in Tibetan), Nam Tso (NMT), Siling Tso (SLT), Co Ngoin Tso (CNT), Tangra Yum Tso (TRYT), Peiku Tso (PKT), Zhari Nam Tso (ZRNMT), Gongzhu Tso (GZT), Rinchen Shup Tso (RQST), Mapam Yum Tso (MPYT), Tuo ji Tso (TJT), and La’ ang Tso (LAT), from west to east (Table S1).

### 2.2. Sample Collection and Analysis

In July 2021, a total of 36 surface sediment samples (0–10 cm) were collected from 12 lakes in Tibet using the Peterson sediment sampler (Figure 1). The sampling sites were in the heart of the lakes, where anthropogenic impacts are low and where the catchment of the lake can be maximized. Three samples were replicated at each site (each sample was spaced 1 m apart), and samples were immediately stored in labeled, self-sealing polyethylene bags and kept in an ice box at 4 °C before being transported back to the laboratory for analysis.

The samples were freeze-dried immediately after the surface sediments were returned to the laboratory, and after drying, they were ground in an agate mortar. The ground samples were passed through a nylon sieve with a pore size of 0.165 mm. Approximately 10 g of sediment powder was obtained for each sample and stored in self-sealing bags. First, 0.5 g of sediment was taken in an ablation tube, and HNO_3_-HCl-HF was added to react overnight. Then, the sample was put it into a microwave digestion apparatus for digestion [39]. The lid was opened at 160 °C to drive out the acid until the color was clear and the solid was gelatinous. The purpose of acid driving is to further release the insoluble and organically complexed metal elements in the sample, and at the same time to drive out the excess acid at high temperature so as to avoid the negative effects of high acid concentration in the digestion matrix on the inductively coupled plasma mass spectrometry (ICP-MS) nebulizer and other components. In a 25 mL volumetric flask, the mixture was transferred and fixed to the scale, which was followed by mixing and filtration through a 0.22 μm filter membrane. The filtered digestion solution was used for the determination of As, Cd, Cr, Cu, Ni, Pb, and Zn by inductively coupled plasma mass spectrometry (Perkin-Elmer NexION, 350B, Waltham, MA, USA). Hg was determined by a DMA80 direct mercury analyzer (Milestone, Milan, Italy). The analyses performed in this study only assessed total metals, and any discussion of bioavailable metals was based on modeling rather than a direct measurement. Total organic carbon (TOC) was determined by the elemental analyzer (EA2400 II), the total phosphorus (TP) was determined by alkali fusion-molybdenum-antimony anti-spectrophotometry, and the pH was determined by a potentiometric method. Sediment particle size was determined by a Malvern particle size analyzer (Mastersizer 3000, Malvern, UK). Prior to the assay, the sediments were pretreated using H_2_O_2_ and HCl solutions with the aim of removing organic matter and carbonates. Granularity was categorized into three size groups: clay (<4 μm), silt (4–63 μm), and sand (63–2000 μm) [40,41].

### 2.3. Quality Assurance and Quality Control

The purity of the reagents used in the sample processing and assay was GR, and all the glassware required for the experiment was soaked in 10% HNO_3_ for 48 h and then washed with ultrapure water and dried in an oven. The correlation coefficients of the calibration curves for all eight heavy metals exceeded 0.999, which was greater than the minimum permissible limit for instrumental analysis. After every ten samples were analyzed, the standard samples were rechecked to ensure measurement accuracy. The quality of the samples was controlled by the national soil composition standards GBW07448 (GSS-19) and GBW07310 (GSD-10), and the recoveries of reference materials ranged from 85.6% to 107.9%, which were lower than the detection limits. All the samples were measured in 3 parallel samples, and both blank and standard samples were set up. The standard deviations between the parallel samples were less than 5%, which was in accordance with the quality control requirements of the analytical experiments.

### 2.4. Risk Assessment

#### 2.4.1. Index of Potential Ecological Risk

Based on the bioavailability and combined effects of heavy metals, Hakanson [42] developed a method for assessing heavy metals’ ecological risk factors. The specific calculation formula is as follows:

(1) $$\mathrm{PERI}=\sum E_{r}^{i}$$

where PERI is the composite potential ecological risk parameter, and $E_{r}^{i}$ is the PERI of metal, indicating the hazardous degree of metal in a lake ecosystem. The $E_{r}^{i}$ was computed using the following equation:

(2) $$E_{r}^{i}=T_{r}^{i}\times \left(C^{i}/C_{n}^{i}\right)$$

where $C^{i}$ is the heavy metal concentration in the sample to be measured, and $C_{n}^{i}$ is the heavy metal background concentration. $T_{r}^{i}$ is the heavy metal toxicity index, and the $T_{r}^{i}$ values of As, Cd, Cr, Cu, Hg, Ni, Pb, and Zn were 10, 30, 2, 5, 40, 2, 5, and 1, respectively [42]. Potential ecological hazards and risk levels are shown in Table S2.

#### 2.4.2. Sediment Quality Guidelines

The SQGs method has been used to assess the biological impacts of heavy metals in sediments [43]. TECs (threshold effect concentrations) and PECs (probable effect concentrations) were assigned to SQGs (Table 1). It is unlikely that adverse biological effects will occur when heavy metal concentrations fall below the TEC, but they are capable of occurring sometimes when concentrations fall between the TEC and the PEC. Heavy metal concentrations higher than the PEC can cause adverse biological effects [43,44].

#### 2.4.3. Index of Toxic Risk

The toxic risk index (TRI) was first developed by Zhang et al. [45] based on the TEC and PEC effect proposed by MacDonald. In addition, there are articles indicating that the TRI was used to reflect the latent toxicity risk of heavy metals to aquatic organisms [46].

(3) $$\mathrm{TRI}=\sum n_{i=1}{\mathrm{TRI}}_{i}=\left\{\left.\left[{\left(C_{i}/{\mathrm{TEC}}_{i}\right)}^{2}+{\left(C_{i}/{\mathrm{PEC}}_{i}\right)}^{2}\right]/2\right\}\right.$$

where TRI_i_ is the toxic risk index of each heavy metal, C_i_ is the content of heavy metal to be measured (mg/kg), TEC_i_ is the threshold effect concentration corresponding to each heavy metal, and PEC_i_ is the probable effect concentration corresponding to each heavy metal. The categories of toxicity risks are shown in Table S3.

#### 2.4.4. Statistical Analysis

Origin 2021 was used to plot the contamination characteristics of the heavy metals in the sediments of the 12 lakes. ArcGIS 10.7 was used for mapping the spatial contamination distribution of heavy metals in sediments of the 12 lakes. Correlations between sediment heavy metals and physicochemical properties were calculated using Pearson analysis in IBM SPSS statistics 19. They were rotated by Kaiser normalized orthogonal axes (varimax) and assigned principal components based on their eigenvalues or cumulative variances. Finally, the calculated Pearson correlation coefficient results are shown by R studio graphing.

## 3. Results and Discussion

### 3.1. Distribution of Other Physical and Chemical Indicators

The physical and chemical factors of the 12 plateau lakes in Tibet studied here are shown in Table S4. The pH of the lake sediments showed a weakly alkaline or alkaline state. Significant differences in TOC for all the lakes were observed, except for Co Ngoin Tso and Tangra Yum Tso. Notably, a large variation in TP content in all lakes was observed, which may be related to anthropogenic inputs of phosphorus. The analysis showed that clay, silt, and sand, representing the variation in sediment particles, differed significantly between the 12 lake sample sites. Specifically, Tangra Yum Tso was dominated by clay, with mean clay, silt, and sand contents of 93.92 ± 1.20%, 2.51 ± 0.22%, and 3.58 ± 1.03%, respectively. Yamzho Yum Tso, Peiku Tso, Gongzhu Tso, Rinchen Shup Tso, Mapam Yum Tso and Tuo ji Tso were dominated by silt, with mean contents of 72.37 ± 0.70%, 77.74 ± 0.58%, 55.64 ± 1.71%, 68.07 ± 0.11%, 50.72 ± 4.75 and 68.31 ± 0.73%. Nam Tso, Siling Tso, Zhari Nam Tso, and La’ ang Tso were dominated by sand, with average sand contents of 62.15 ± 0.08%, 87.82 ± 0.04%, 72.68 ± 0.04%, 67.46 ± 0.18%, and 60.07 ± 0.17%, respectively.

### 3.2. Concentration of Heavy Metals in Surface Sediment

Heavy metals in sediments from the 12 Tibetan lakes were analyzed descriptively, and the results are shown in Table 1. The ranges of heavy metal concentrations in the 12 lake samples were As 6.59–331.87 mg/kg, Cd 0.04–0.32 mg/kg, Cr 17.25–426.94 mg/kg, Cu 3.24–26.17 mg/kg, Hg 0.01–0.06 mg/kg, Ni 6.95–211.86 mg/kg, Pb 7.58–49.41 mg/kg, and Zn 29.67–91.01 mg/kg. The coefficient of variation (CV) was used to indicate the degree of spatial variability of heavy metals in surface sediments [47]. The CVs of As, Cd, Cr, Hg, and Ni were 169.72%, 61.19%, 133.81%, 55.45%, and 151.59%, respectively, which indicated high variation. Lake sediments might be polluted by As, Ni, Cr, Cd, and Hg to varying degrees.

A comparison of heavy metal contents in surface sediments of lakes on the Tibetan Plateau with other regions is shown in Table 1. The results showed that the As content in 58% of the lakes in this study exceeded the soil background value [48], ranging from 1.45 to 19.65 times the soil background value, which may be related to the As-rich host rock widely distributed in the Tibetan Plateau [32]. The As content of the twelve plateau lakes in this study was significantly higher than the crustal abundance: even in Nam Tso, which had the lowest As content, the values were 3.47 times the Chinese abundance and 2.99 times the global crustal abundance, respectively. However, the As concentration in Gongzhu Tso was 174.67 times the Chinese abundance and 150.85 times the global crustal abundance. It is a carcinogen that has been identified as a potential threat to aquatic ecosystems [49]. However, the contents of various heavy metals in the surface sediments of Siling Tso and Co Ngoin Tso extracted from northern Tibet were lower than those of other lakes as a whole. This may be attributed to the fact that Siling Tso and its surrounding areas are national protected areas in China, coupled with the fact that northern Tibet is mainly dominated by the Tibetan people, who are in awe of nature in their daily lives and are more attentive to the protection of ecological environments on which they depend for their lives in terms of their human–nature relationship [50]. The existence of these combined reasons makes the heavy metal content in the surface sediments of the two plateau lakes in northern Tibet close to the background value of the soil in the region. In addition, Nam Tso is similar to the above, with heavy metal levels in surface sediments close to soil background values. Recent studies have likewise reported that the origin of most elements in surface water sediments in the southern Tibetan Plateau is mainly related to natural diagenesis, with little contamination [49]. In addition, there are some discrepancies between the Tibetan soil background values selected for this study and the crustal element abundances in China and globally, resulting in that such discrepancies may be attributed to the fact that crustal element abundances are determined at specific times and regions [51,52]. Heavy metals in the surface and crustal environments of different regions are affected by factors such as topography and climate and undergo a complex process of transport and transformation, resulting in spatial differences between heavy metal soil background values and crustal abundances [53]. Some studies have also shown that the background values of soil samples are related to the different geochemical behaviors of leaching, migration, and accumulation of elements in soil in different regions and zones [54]. It is interesting to see that Cd, Cu, Hg, Pb, and Zn are all below or close to the Chinese and global crustal abundances. A one-way analysis of variance (ANOVA) and Duncan’s test were used to analyze the variance of eight heavy metals (As, Cd, Cr, Cu, Hg, Ni, Pb, and Zn) in the surface sediments extracted from the 12 lakes (Table S5). It is noteworthy that the lakes with metal concentrations exceeding the background values were mainly concentrated in five lakes in southwest Tibet (Mapam Yum Tso, Tuo ji Tso, Rinchen Shup Tso, Gongzhu Tso, and La’ ang Tso). The ANOVA results showed that both Mapam Yum Tso and La’ ang Tso had significantly greater Cr and Ni concentrations than the remaining lakes (*p* < 0.05) and crustal abundances in China. In addition, the As content in Mapam Yum Tso was also significantly higher than in the rest of the lakes (*p* < 0.05), and the As content was three times higher than that in La’ ang Tso, which is similar to the findings in a recent study [55]. Compared with the average lake sediment values (ALS) of Chinese lakes, the heavy metal contents in the surface sediments of all lakes were lower than or close to the ALS, except for As, Cr, and Ni.

The percentage of lakes with elemental contents in the sediment above the PEC value is shown in Table 1. The highest contamination level was found in lake sediments for As, Cr, and Ni. Among these, As content exceeded the PEC value in 25% of the lake sediments, namely in Gongzhu Tso, Rinchen Shup Tso, and La’ ang Tso. This was followed by Cr and Ni, with approximately 16.66% of the lake sediments containing higher levels of Cr and Ni than the corresponding PEC values. Notably, the lakes with Cr and Ni levels exceeding the PEC values were Mapam Yum Tso and La’ ang Tso. The results suggested that Cr and Ni may often cause adverse biological effects in Mapam Yum Tso and La’ ang Tso. Considering the lower Cd, Cu, Hg, Pb, and Zn concentrations in lake sediment than in the PEC value, it is expected that none of these metals would be harmful to biological organisms.

**Table 1:** Descriptive statistics of heavy metals in surface sediments of 12 plateau lakes in Tibet (mg/kg).

	As	Cd	Cr	Cu	Hg	Ni	Pb	Zn
Yamzho Yum Tso	24.44 ± 2.47	0.05 ± 0.01	37.60 ± 10.90	26.17 ± 6.89	0.035 ± 0.001	17.31 ± 0.76	11.95 ± 1.64	39.43 ± 4.12
Nam Tso	6.59 ± 1.79	0.13 ± 0.02	27.87 ± 1.84	8.48 ± 1.24	0.016 ± 0.001	11.05 ± 0.34	21.41 ± 0.87	29.67 ± 12.43
Siling Tso	16.79 ± 2.64	0.13 ± 0.02	54.68 ± 3.13	14.74 ± 2.19	0.060 ± 0.005	20.20 ± 2.22	23.70 ± 0.11	58.67 ± 2.97
Co Ngoin Tso	11.72 ± 1.26	0.05 ± 0.01	24.47 ± 0.53	6.20 ± 0.62	0.010 ± 0.001	9.91 ± 0.65	20.20 ± 1.04	39.13 ± 10.84
Tangra Yum Tso	27.29 ± 0.92	0.18 ± 0.01	52.58 ± 4.99	19.55 ± 0.60	0.019 ± 0.001	19.96 ± 0.36	49.41 ± 2.63	91.01 ± 3.08
Peiku Tso	15.34 ± 4.02	0.12 ± 0.01	49.66 ± 7.76	10.08 ± 0.92	0.033 ± 0.004	14.88 ± 0.14	21.36 ± 0.26	45.62 ± 6.49
Zhari Nam Tso	28.79 ± 4.42	0.10 ± 0.01	17.25 ± 1.98	3.24 ± 1.64	0.019 ± 0.001	6.95 ± 1.06	18.11 ± 0.74	48.32 ± 3.40
Gongzhu Tso	331.87 ± 21.63	0.09 ± 0.01	63.15 ± 8.35	20.39 ± 3.26	0.014 ± 0.001	22.61 ± 0.95	18.09 ± 0.17	40.79 ± 4.99
Rinchen Shup Tso	38.16 ± 7.29	0.25 ± 0.02	22.35 ± 1.55	18.17 ± 5.02	0.011 ± 0.001	8.53 ± 0.46	38.44 ± 1.56	90.58 ± 20.03
Mapam Yum Tso	28.69 ± 6.42	0.09 ± 0.01	142.96 ± 17.66	15.54 ± 5.26	0.028 ± 0.004	61.82 ± 8.07	29.17 ± 1.05	44.25 ± 7.93
Tuo ji Tso	15.11 ± 4.02	0.32 ± 0.01	57.03 ± 7.76	23.39 ± 0.92	0.042 ± 0.004	28.68 ± 0.14	27.54 ± 0.26	81.74 ± 6.49
La’ ang Tso	63.11 ± 3.71	0.04 ± 0.01	426.94 ± 11.72	11.74 ± 1.45	0.021 ± 0.002	211.86 ± 23.31	7.58 ± 0.36	33.42 ± 7.93
Maximum	331.87	0.32	426.94	26.17	0.06	211.86	49.41	91.01
Minimum	6.59	0.04	17.25	3.24	0.01	6.95	7.58	29.67
CV	169.72%	61.19%	133.81%	45.64%	55.45%	151.59%	45.26%	39.31%
TEC	9.79	0.99	43.4	31.6	0.18	22.7	35.5	121
PEC	33	4.98	111	149	1.06	48.6	128	459
<TEC(%)	8.33%	100.00%	41.67%	100.00%	100.00%	75.00%	83.33%	100.00%
≥TEC < PEC(%)	66.67%	-	41.67%	-	-	8.33%	16.67%	-
>PEC(%)	25.00%	-	16.66%	-	-	16.67%	-	-
Soil background value in Tibet [48]	16.8	0.078	68.1	19.6	0.02	28.7	27.9	71.1
China’s crustal abundance [52]	1.90	0.05	63.00	38.00	0.02	57.00	15.00	86.00
Global crustal abundance [52]	2.20	0.20	73.50	63.00	0.45	89.00	12.00	94.00
Average value of lake sediment in China [56]	12.1	0.194	85	31.7	0.053	36.8	31	88

### 3.3. Spatial Distribution of Heavy Metals in Surface Sediments

The highest values of different metal elements were found in various lakes, and the highest value of As was seen in Gongzhu Tso, with a value of 331.87 mg/kg, while the highest value of Cd was 0.32 mg/kg in Tuo ji Tso. The highest values of Cr and Ni were 426.94 mg/kg and 211.86 mg/kg, respectively, both of which were found in La’ ang Tso. Both Yamzho Yum Tso and Siling Tso contained the highest levels of Cu and Hg at 26.1 mg/kg and 0.06 mg/kg, respectively. In Tangra Yum Tso, Pb and Zn were the highest, at 49.41 mg/kg and 91.01 mg/kg, respectively. It should be emphasized that the concentration of As in Tangra Yum Tso, Zhari Nam Tso, Gongzhu Tso, Rinchen Shup Tso, and Mapam Yum Tso and the concentrations of As, Cr, and Ni in La’ ang Tso were all higher than the soil environment quality risk control standards for soil contamination of agriculture land [57]. Among the eight heavy metals measured, the concentrations of all the heavy metals were close to or lower than the background values of Tibetan soil, except for As, Cd, Cr, Hg, and Ni, which were higher than the SBT. Remarkably, Gongzhu Tso had the highest content of As in the 12 lakes, which was nineteen times higher than the SBT. The content of As from the lake sediments on the Tibetan Plateau is high due to the distribution of As-rich parent rocks such as shale, schist, and micrite [32].

**Figure 2:**
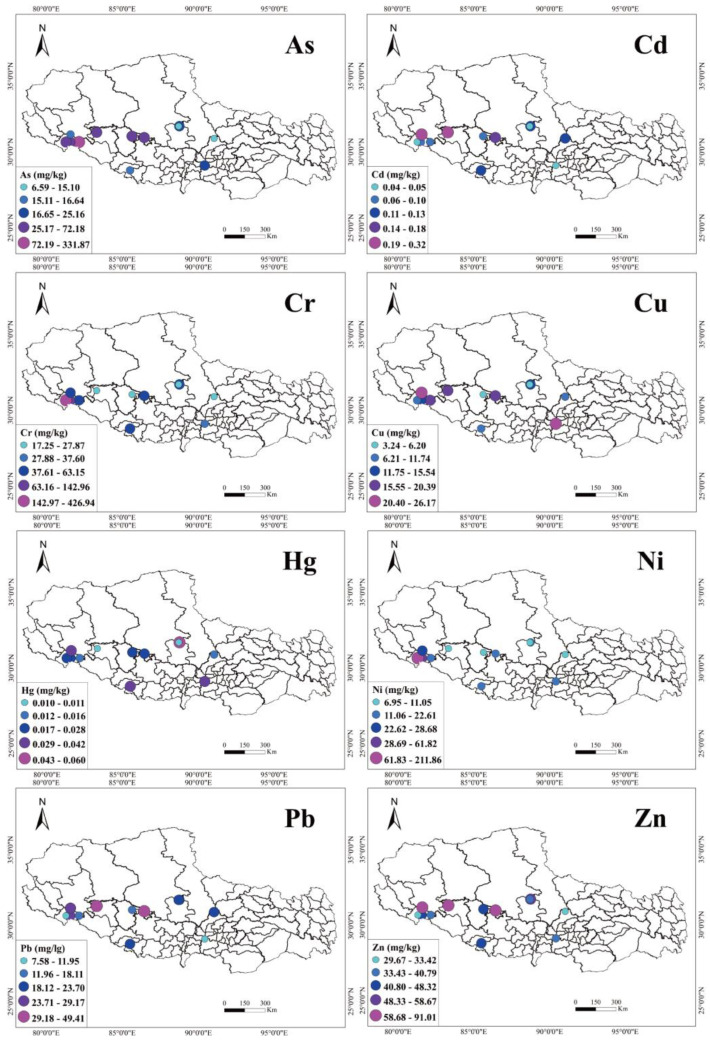
Spatial distribution of heavy metals in surface sediment of 12 plateau lakes in Tibet.

The spatial distribution of each heavy metal has no significant distribution pattern (Figure 2). The basic climatic characteristics of the Qinghai–Tibet Plateau are cold and arid, low precipitation, and intense evaporation, so snow- and ice-melt water and groundwater are the primary forms of lake recharge [58]. Due to the distribution of As-rich shales in Tibet, high-temperature water–rock interactions can readily dissolve As in As-bearing minerals and expel them as gaseous fluids released by geothermal activity, which can be fed into lakes via rivers [36]. Still, these are mostly closed lakes where evaporation and the concentration of lake water and subsequent sediment adsorption can lead to further elevated As levels [36].

### 3.4. Risk Assessment

#### 3.4.1. Toxic Risk Index Assessment

**Figure 3:**
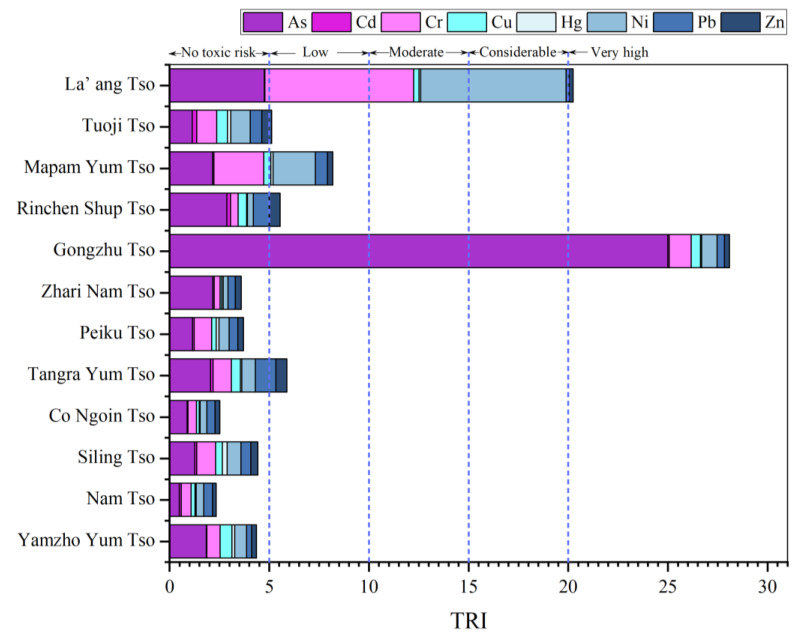
The TRI of heavy metal in surface sediment of 12 plateau lakes in Tibet.

The TRI of sediments in the research area ranged from 2.34 (Nam Tso) to 28.09 (Gongzhu Tso), with a mean value of 7.84. Only Gongzhu Tso (28.09) and La’ ang Tso (20.25) showed high TRI values (Figure 3), which indicated that heavy metals from these two lakes might pose a high toxicity risk to aquatic organisms. However, Tangra Yum Tso (5.88), Rinchen Shup Tso (5.55), Mapam Yum Tso (8.19), and Tuo ji Tso (5.12) presented low toxicity risks. In addition, the remaining lakes with TRI values of less than 5 were considered, as the heavy metal content in the lakes may not pose a toxic risk to aquatic organisms. As contributed the most to the total TRI value for Gongzhu Tso. For La’ ang Tso, Cr contributed the most to the overall TRI value for La’ ang Tso, followed by Ni and As (Figure 3).

#### 3.4.2. Potential Ecological Risk Assessment

The potential ecological risk factors ($E_{r}^{i}$) are shown in Table S6. The pollution degree of eight heavy metals decreased in the following order: Hg (51.10) > Cd (50.50) > As (30.15) > Ni (6.30) > Pb (4.29) > Cu (3.78) > Cr (2.39) > Zn (0.75). The $E_{r}^{i}$ values of Cr, Ni, Zn, Pb, Ni, and Cu in the surface sediments of the 12 lakes in this study are all below 40, which was classed as a low degree of potential ecological risk. For Cd, the $E_{r}^{i}$ in Nam Tso, Siling Tso, Tangra Yum Tso, and Peiku Tso posed a moderate risk, with $E_{r}^{i}$ between 40 and 80. In addition, the $E_{r}^{i}$ values for Cd in Rinchen Shup Tso (97.94) and Tuo ji Tso (123.45) were also at a considerable risk level. For Hg, the $E_{r}^{i}$ values in Yamzho Yum Tso, Peiku Tso, Mapam Yum Tso, and La’ ang Tso appear to pose a moderate risk, with $E_{r}^{i}$ values between 40 and 80. However, the $E_{r}^{i}$ values for Hg in Tuo ji Tso (83.33) and Siling Tso (119.87) posed a considerable risk.

**Figure 4:**
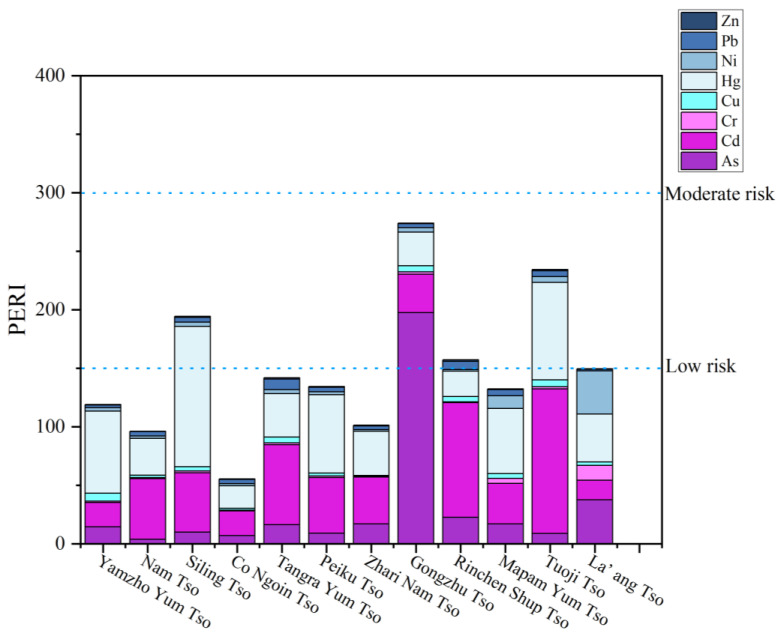
The PERI of heavy metals in surface sediment of 12 plateau lakes in Tibet.

The potential ecological risk index of the 12 Tibetan lakes varied between 55.58 and 274.07, with an average of 149.25. A medium-risk category was assigned to 33.3% of the lakes in this study (Figure 4), namely Siling Tso (194.41), Gongzhu Tso (274.07), Rinchen Shup Tso (157.20), and Tuo ji Tso (234.50). The ecological risk level of Gongzhu Tso is the highest. This may be because Gongzhu Tso has a semi-arid climate, and the high evaporation rate may increase the enrichment of heavy metals in the lake sediment [59]. Meanwhile, a national road has been constructed north of Gongzhu Tso, and the growth of tourism in Tibet in recent years may be the reason for its high PERI value. Related studies have shown that the majority of lake ecosystems are vulnerable to human activities, such as land development and industrial production [60]. In addition, studies from Wuliangsu Lake [61] and Aibi Lake [62] have also shown that human mining activities affect the enrichment of heavy metals in lake sediments. Except for the four lakes mentioned above, all the lakes fall into the low-risk category. Nationally, the PERI values for the sediments of the 12 Tibetan plateau lakes were all lower than the average PERI value of Chinese lake sediments (PERI = 327) [61].

### 3.5. Source Identification of Heavy Metals in the Plateau Lakes in Tibet

**Figure 5:**
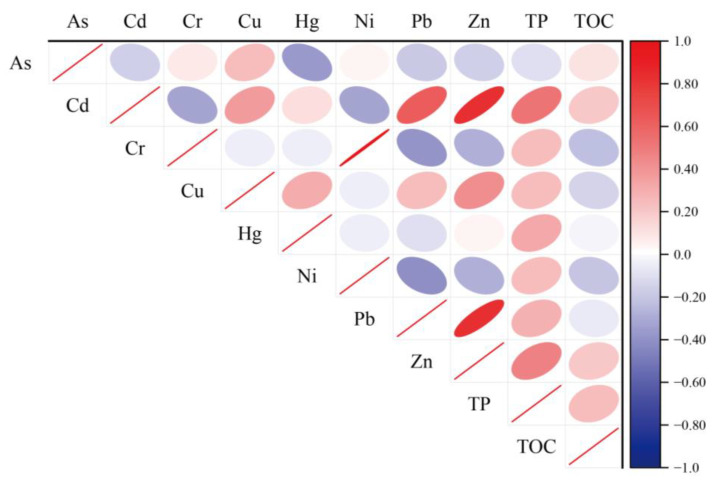
Pearson correlation analysis of heavy metals and TOC and TP in surface sediment of plateau lakes in Tibet (*p* < 0.05).

**Figure 6:**
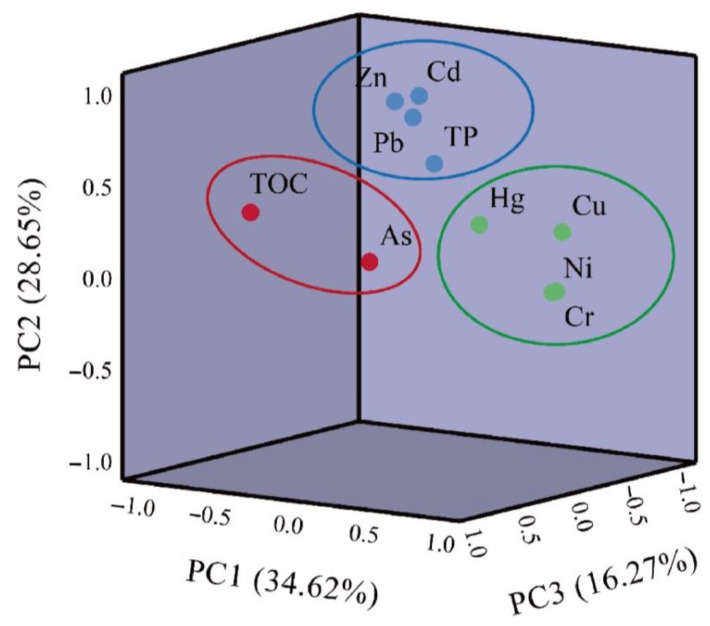
PCA results of heavy metals and TOC and TP in surface sediments of plateau lakes in Tibet.

A correlation analysis (Figure 5) and principal component analysis (PCA) (Figure 6) were used to identify the potential sources of heavy metals in the sediments [63]. The origin and transport of metallic elements can be reflected by correlations between heavy metal concentrations [64]. The principal component analysis of heavy metals in the sediments is shown in Table S7. In this PCA based on the criterion of Kaiser eigenvalues greater than 1, three principal component factors with contributions of 34.62%, 28.65%, and 16.27% were used. The three component factors together account for 79.53% of the variance, which was sufficient to describe the bulk of information sources on all heavy metals. Figure 6 shows the loading diagram of the main components of heavy metals in the sediments. With a Kaiser–Meyer–Olkin value of 0.530 (>0.5) and Bartlett’s test p of 0.001 (<0.05), the results of the principal component analysis are reliable and suitable for source identification [65].

The first principal component (PC1) explained 34.62% of the total variance. The main characteristics of PC1 were the weak positive loading of Hg and the strong negative loading of Ni, Cr, and Cu. In addition, there was a significant positive correlation between Cr, Cu, and Ni (Figure 5), indicating that Cr, Cu, Hg, and Ni exhibit the same transport behaviors, have similar levels of contamination, and have common sources. Minor contamination with Cr, Ni, Hg, and Cu was found in some lakes due to the above contamination assessment. Therefore, it can be inferred that the first principal component is related to human activities. Previous studies have suggested that Hg may be associated with atmospheric pollution caused by long-range transport following industrial emissions [33], and that metals such as Cu and Ni are released during electroplating [66]. Pesticides and fertilizers used in agriculture may cause Cu to enter lakes through surface runoff, and these chemicals may eventually accumulate in the sediment [67]. It can thus be interpreted that PC1 represents both agricultural and industrial activities.

The second principal component (PC2) explained 28.65% of the total variance, which had a higher factor loading of Zn, Cd, Pb, and TP (>0.65), and Cd, Pb, and Zn were significantly positively correlated (Figure 5). The sampled lakes were distributed around Qinghai–Tibet highways, and related research shows that Cd, Pb, and Zn are mainly released through the incomplete combustion of automotive fuels and tire wear and tear [68,69]. Previous studies of atmospheric heavy metal deposition in Tibet have shown that Cd and Pb can condense and settle down after long-range transport from South Asia [70]. In addition, TP showed a weak positive correlation with Cd, Pb, and Zn, indicating that anthropogenic impacts have led to TP enrichment in lake sediments on the Tibetan Plateau [71]. Thus, PC2 may be affected by a combination of traffic and atmospheric transport.

PC3 explained 16.27% of the total variance, with a higher factor loading of As and TOC (>0.7), suggesting that the As accumulation in the surface sediment differs from the other heavy metals. Heavy metal distribution in sediment has been largely influenced by TOC [72]. As and TOC were, however, weakly correlated in this study. Organic carbon is a ligand and adsorption carrier for heavy metals and can affect heavy metals through a series of reactions, such as adsorption, complexation, and precipitation [72,73]. Additionally, the variation in TOC content was reported to be mainly influenced by regional vegetation [74]. Further, several reports point to widespread distributions of As-rich schist, micrite, and shale rocks on the Qinghai–Tibet Plateau [32,75,76,77]. Meanwhile, geothermal activities influence As elements [36,58]. Therefore, PC3 primarily indicated natural sources.

## 4. Conclusions

In sediments from 12 lakes in Tibet, the mean values of Cr, Hg, As, Cd, and Ni exceeded the Tibetan soil background values, but most values were less than the TEC values. Regardless of geographical location, the TRI indicated that most of the lake sediments in Tibet were relatively clean, with only Gongzhu Tso being at high risk of As toxicity and La’ ang Tso being at moderate risk of As, Cr, and Ni toxicity. At the same time, different levels of Hg pollution occurred in the lakes due to socio-economic development. The PERI likewise corroborates the above pollution index analysis results. Therefore, it is recommended that the relevant authorities strengthen the monitoring of As, Cd, Ni, and Hg in lake sediments in Tibet.

Using a principal component analysis, we inferred that Cu, Cr, Hg, and Ni were mainly derived from industry and agriculture; Cd, Pb, and Zn from transport and atmospheric transfer; and As mainly from geothermal activity and As-rich shale and schist. At the same time, TOC indirectly contributes to the deposition of elemental As.

Overall, the present study provides a systematic evaluation of the contamination levels of heavy metals in the surface sediments of 12 plateau lakes in Tibet, which can help in formulating policies and taking appropriate measures for the management and prevention of toxic elemental contamination in the ecosystems of these lakes. The study suggests that local governments should strengthen pollution monitoring along with economic development, pay more attention to the impact of heavy metals on aquatic ecosystems, and enhance source control and public awareness.

## Data Availability

Some or all data, models, or code that support the findings of this study are available from the corresponding author upon reasonable request.

## Supplementary Materials

The following supporting information can be downloaded at: https://www.mdpi.com/article/10.3390/toxics11100804/s1, Table S1: Geographical location and basic characteristics of 12 studied lakes in Tibet; Table S2: Indices and grades of potential ecological risk; Table S3: Indices and grades of toxic risk; Table S4: Variation characteristics of sediment physicochemical properties of 12 studied lakes in Tibet; Table S5: The contents of heavy metal (mg/kg) in 12 studied lake sediments from Tibet; Table S6: Potential ecological risk index of heavy metals in sediments from 12 lakes in Tibet; Table S7: Rotated component matrix of heavy metal principal component analysis loadings in sediments from 12 lakes in Tibet.
