# Supplementary material for: Heavy Metals in Surface Sediment of Plateau Lakes in Tibet, China: Occurrence, Risk Assessment, and Potential Sources
Source: Toxics. 2023 Sep 23;11(10):804. doi: 10.3390/toxics11100804 (PMC10610607; doi:10.3390/toxics11100804)
Supplement: Supplementary file 1 [file toxics-11-00804-s001.zip › toxics-2599582-supplementary.pdf]

SUPPORTING INFORMATION

Heavy Metals in Surface Sediment of Plateau Lakes in Tibet, China: Occurrence, Risk Assessment, and Potential Sources

Qiongyuan Su, Asfandyar Shahab, Liangliang Huang, Muhammad Ubaid Ali, Yanan Cheng, Jiahuan Yang, Hao Xu, Zhicheng Sun, Qi Zou, Zhongbing Chen and Bin Kang

Table S1. Geographical location and basic characteristics of 12 studied lakes in Tibet.

Lake name	L/ (°)	N/ (°)	Alt/(m)	pH	Lake area/(km^2)	Watershed area/(km^2)
Yamzho Yum Tso	90.4182	29.1137	4419	8.57	592	6100
Nam Tso	91.0314	30.8900	4692	8.74	1981	10610
Siling Tso	88.7528	31.6953	4525	8.81	1640	45530
Co Ngoin Tso	88.7075	31.6900	4526	8.36	61.3	1019.7
Tangra Yum Tso	86.4466	30.9506	4528	8.81	835.3	9059
Peiku Tso	85.5106	28.7967	4558	8.72	270.7	2820
Zhari Nam Tso	85.6453	31.0344	4597	8.98	1023	20341
Gongzhu Tso	82.1600	30.6539	4756	8.82	66.2	849.8
Rinchen Shup Tso	83.3233	31.2880	4730	8.89	187.1	2472
Mapam Yum Tso	81.5608	30.6511	4552	9.08	412.1	4560
Tuoji Tso	81.6042	31.1497	5340	7.49	—	—
La′ ang Tso	81.3075	30.6547	4572	8.59	242.9	2551.5

Table S2. Indices and grades of potential ecological risk.

E_r^i	Grades of ecological risk for a single metal	RI	Grades of potential ecological risk to the environment
$E_r^i \leq 40$	Low risk	RI $<$ 150	Low risk
$40 \leq E_r^i < 80$	Moderate risk	$150 \leq$ RI $<$ 300	Moderate risk
$80 \leq E_r^i < 160$	Considerable risk	$300 \leq$ RI $<$ 600	Considerable risk
$160 \leq E_r^i < 320$	High risk	RI $\geq$ 600	High risk
$E_r^i \geq 320$	Very high risk		

Table S3. Indices and grades of toxic risk.

TRI	Contamination grade
TRI ≤ 5	no toxic risk
5 < TRI ≤ 10	low toxic risk
10 < TRI ≤ 15	moderate toxic risk
15 < TRI ≤ 20	considerable toxic risk
20 < TRI	very high toxic risk

Table S4. Variation characteristics of sediment physicochemical properties of 12 studied lakes in Tibet.

Lake name	pH	TP	TOC	Clay	Silt	Sand
Yamzho Yum Tso	8.06±0.04[a]	139±62.88[a]	601±2.49[a]	24.61±0.54[a]	72.37±0.70[a]	3.02±0.17[a]
Nam Tso	8.07±0.05[a]	148±71.36[a]	300±0.47[b]	3.99±1.25[bc]	34.41±0.87[b]	61.60±1.16[b]
Siling Tso	9.42±0.02[b]	478±96.82[bcd]	754±1.63[c]	3.60±0.62[b]	9.84±0.36[c]	86.57±0.97[c]
Co Ngoin Tso	8.67±0.04[c]	194±10.66[a]	373±0.82[d]	4.83±0.48[bc]	22.40±0.88[d]	72.77±0.41[d]
Tangra Yum Tso	9.22±0.04[eg]	540±81.45[cde]	368±6.53[d]	93.92±1.20[d]	2.51±0.22[e]	3.58±1.03[a]
Peiku Tso	7.85±0.04[d]	233±36.54[a]	263±2.45[e]	14.66±0.79[e]	77.74±0.58[f]	7.60±0.85[e]
Zhari Nam Tso	7.89±0.02[d]	456±101.48[cbd]	2016±8.49[f]	3.78±1.25[bc]	31.11±1.42[g]	65.11±1.84[f]
Gongzhu Tso	9.48±0.03[b]	302±93.53[ab]	838±9.79[g]	5.37±0.40[c]	55.64±1.71[h]	38.99±2.08[g]
Rinchen Shup Tso	8.71±0.03[c]	281±53.10[ad]	1027±12.24[h]	27.78±0.67[f]	68.07±0.11[i]	4.15±0.77[a]
Mapam Yum Tso	9.17±0.03[e]	676±79.58[e]	543±0.82[i]	8.60±0.44[g]	50.72±4.75[j]	40.68±4.36[g]
Tuoji Tso	6.90±0.02[f]	956±48.86[f]	965±5.71[j]	8.69±0.43[g]	68.31±0.73[k]	22.99±0.30[h]
La′ ang Tso	9.29±0.04[g]	498±187.28[ceg]	465±0.82[k]	2.82±0.43[b]	36.78±0.11[b]	60.40±0.46[b]

[abcdefghijk] Values in each column with the same letters are not significantly different (Bonferroni) between the 12-lake sediment in Tibet ($P > 0.05$).

Table S5. The contents of heavy metal (mg/kg) in 12 studied lake sediments from Tibet.

Lake name	As	Cd	Cr	Cu	Hg	Ni	Pb	Zn
Yamzho Yum Tso	24.44[adg]	0.05[dgi]	37.60[ae]	26.17[ad]	0.04[a]	17.31[abc]	11.95[abh]	39.43[a]
Nam Tso	6.59[b]	0.13[agd]	27.87[ade]	8.48[ad]	0.02[a]	11.05[ac]	21.41[bceh]	29.67[ab]
Siling Tso	16.79[abdeh]	0.13[abdefg]	54.68[ade]	14.74[ad]	0.06[a]	20.20[abc]	23.70[bcde]	58.67[a]
Co Ngoin Tso	11.72[ab]	0.05[defg]	24.47[ae]	6.20[cd]	0.01[a]	9.91[ac]	20.20[abceh]	39.13[ab]
Tangra Yum Tso	27.29[dg]	0.18[aef]	52.58[ade]	19.55[ab]	0.02[a]	19.96[abc]	49.41[cdefgj]	91.01[b]
Peiku Tso	15.34[abdeh]	0.12[agd]	49.66[ade]	10.08[cd]	0.03[a]	14.88[abc]	21.36[bcde]	45.62[ab]
Zhari Nam Tso	28.79[eg]	0.10[adefg]	17.25[a]	3.24[ad]	0.02[a]	6.95[cd]	18.11[bch]	48.32[a]
Gongzhu Tso	331.87[f]	0.09[agd]	63.15[ed]	20.39[ad]	0.01[a]	22.61[ab]	18.09[hgi]	40.79[a]
Rinchen Shup Tso	38.16[g]	0.25[abc]	22.35[ae]	18.17[ad]	0.01[a]	8.53[abd]	38.44[di]	90.58[ab]
Mapam Yum Tso	28.69[hg]	0.09[dfg]	142.96[b]	15.54[ad]	0.03[a]	61.82[e]	29.17[de]	44.25[ab]
Tuo ji Tso	15.11[abdeh]	0.32[adg]	57.03[d]	23.39[ad]	0.04[a]	28.68[b]	27.54[bcdf]	81.74[ab]
La′ ang Tso	63.11[c]	0.04[di]	426.94[c]	11.74[ad]	0.02[a]	211.86[f]	7.58[afj]	33.42[ab]

Note: The data in the table are mean values (n=3). Different lower-case letters in the same column indicate significant differences (P<0.05) for the same heavy metal in different lake sediments.

Table S6. Potential ecological risk index of heavy metals in sediments from 12 lakes in Tibet.

Lake name	E_r^i								RI
	As	Cd	Cr	Cu	Hg	Ni	Pb	Zn	
Yamzho Yum Tso	14.55	20.90	1.10	6.68	70.13	3.02	2.14	0.55	119.07
Nam Tso	3.92	51.82	0.82	2.16	31.40	1.92	3.84	0.42	96.31
Siling Tso	9.99	50.59	1.61	3.76	119.87	3.52	4.25	0.83	194.41
Co Ngoin Tso	6.98	21.07	0.72	1.58	19.33	1.73	3.62	0.55	55.58
Tangra Yum Tso	16.24	68.50	1.54	4.99	37.07	3.48	8.85	1.28	141.96
Peiku Tso	9.13	47.39	1.46	2.57	66.80	2.59	3.83	0.64	134.42
Zhari Nam Tso	17.14	39.90	0.51	0.83	37.93	1.21	3.25	0.68	101.43
Gongzhu Tso	197.54	32.92	1.85	5.20	28.80	3.94	3.24	0.57	274.07
Rinchen Shup Tso	22.71	97.94	0.66	4.64	21.60	1.49	6.89	1.27	157.20
Mapam Yum Tso	17.08	34.66	4.20	3.96	55.87	10.77	5.23	0.62	132.39
Tuoji Tso	8.99	123.45	1.67	5.97	83.33	5.00	4.93	1.15	234.50
La′ ang Tso	37.56	16.81	12.54	2.99	41.07	36.91	1.36	0.47	149.72

Table S7. Rotated component matrix of heavy metal principal component analysis loadings in sediments from 12 lakes in Tibet.

Heavy metal	PC1	PC2	PC3
Ni	**0.985**	-0.026	0.061
Cr	**0.981**	-0.027	0.017
Cu	**0.762**	0.222	-0.350
Hg	**0.531**	0.304	0.099
Zn	-0.034	**0.919**	0.089
Cd	-0.154	**0.879**	-0.311
Pb	-0.308	**0.723**	-0.474
TP	0.396	**0.657**	0.331
As	0.269	0.169	**0.759**
TOC	-0.563	0.346	**0.690**
Eigenvalue	3.462	2.864	1.627
Variance (%)	34.62	28.64	16.27
Accumulative (%)	34.62	62.26	79.53
